# Molecular descriptor data explain market prices of a large commercial chemical compound library

**DOI:** 10.1038/srep28521

**Published:** 2016-06-23

**Authors:** Jaroslaw Polanski, Urszula Kucia, Roksana Duszkiewicz, Agata Kurczyk, Tomasz Magdziarz, Johann Gasteiger

**Affiliations:** 1 Institute of Chemistry, University of Silesia, 9 Szkolna Street, 40-006 Katowice, Poland; 2 Institute of Automatic Control, Silesian University of Technology, 16 Akademicka Street, 44-100 Gliwice, Poland; 3 Biotechnology Centre, Silesian University of Technology, 8 Krzywoustego Street, 44-100 Gliwice, Poland; 4 Computer-Chemie-Centrum, University of Erlangen-Nuernberg, Naegelsbachstrasse 25, 91052 Erlangen, Germany

## Abstract

The relationship between the structure and a property of a chemical compound is an essential concept in chemistry guiding, for example, drug design. Actually, however, we need economic considerations to fully understand the fate of drugs on the market. We are performing here for the first time the exploration of quantitative structure-economy relationships (QSER) for a large dataset of a commercial building block library of over 2.2 million chemicals. This investigation provided molecular statistics that shows that on average what we are paying for is the quantity of matter. On the other side, the influence of synthetic availability scores is also revealed. Finally, we are buying substances by looking at the molecular graphs or molecular formulas. Thus, those molecules that have a higher number of atoms look more attractive and are, on average, also more expensive. Our study shows how data binning could be used as an informative method when analyzing big data in chemistry.

What is the market value of a given molecule? How do economy and chemistry contribute to the price of a chemical compound and how can we measure these different contributions? The relationship between the structure and property is an essential concept in chemistry and this method is an important decision-making guide, for example, in drug design. Actually, however, we need economic considerations to fully understand the fate of drugs on the market.

Economy controls all aspects of human behavior. In particular, in science it is probably as important as science itself in deciding research opportunities and directions as well as business R&D decisions. Market balances prices through demand and supply. Does this also work on the molecular market? How important is the contribution of the composition of a chemical to its price? Is there any influence of molecular complexity on the price and how can this be measured? Actually the prices of chemical substances have never been analyzed as a function of structure descriptors and the only analysis relates the prices of a small set of 300 common chemicals to the frequency with which they were recorded as a product or as a substrate in the Beilstein database^1^,^2^.

Here we analyzed the chemical and economic data for a chemical library registering over 2.2 million compounds, thus providing a wide representation of the chemical space, especially if we remember that the actual availability of a majority of the 100 million compounds registered in CAS is limited. These compounds in CAS were synthesized, then few properties were registered, but then these compounds usually are not on hand any more, unless their synthesis would be repeated. To the best of our knowledge this is the first study between economic and molecular descriptors that has ever been reported.

## Results

**Figure 1:**
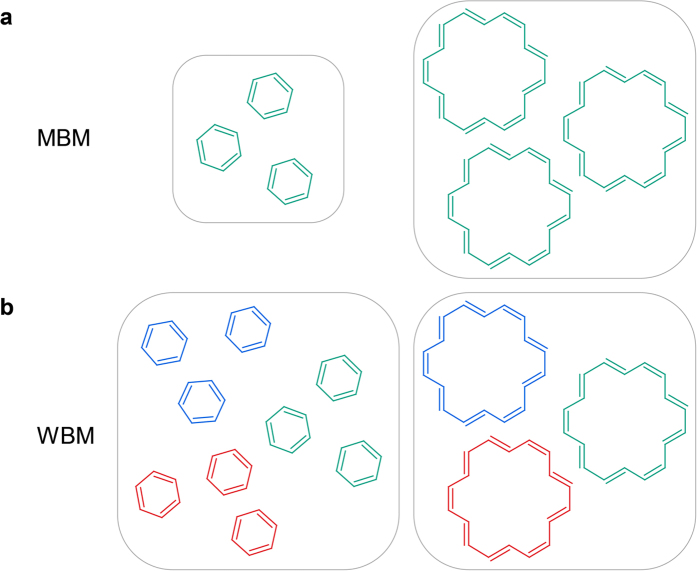
Comparison of the weight (WBM) vs. molar based metrics (MBM). (**a**) MBM: the Avogadro number of molecules (higher weight for higher MW molecules); (**b**) WBM: a unit weight can contain either a larger number of lower MW molecules or a lower number of the higher MW molecules. Thus, the ratio of the benzene to [18]annulene molecules in the same weight unit will amount to 1 to 3 in the WBM.

Unlike in chemistry where we are familiar with the molar idea of molecular behavior, the market sells substances generally on a weight basis, similar to other goods. Thus, a typical *economic property* is the price for a certain weight unit given in $/g. In Fig. 1 we compare the idea of weight based metrics (WBM) vs. the molar based metric (MBM). MBM counts the number of molecules. If we, however, normalize by the amount of weight, then the atoms of the same total atomic weights can be arranged either into a single molecule of higher molecular weight (MW) or several smaller molecules of the lower MW. For example, we can arrange from a certain amount of matter three molecules of a single benzene molecule C_6_H_6_ (MW = 78) but also a single [18]annulene molecule C_18_H_18_ (MW = 3^*^78). It is widely true that the larger is a molecule the more complex it is. Thus, MW could be seen as the simplest but rough, measure of molecular complexity. A question arises now what is the pricing for the WBM. Is it based on the molecule size (MW) or on the number of molecules delivered?

**Figure 2:**
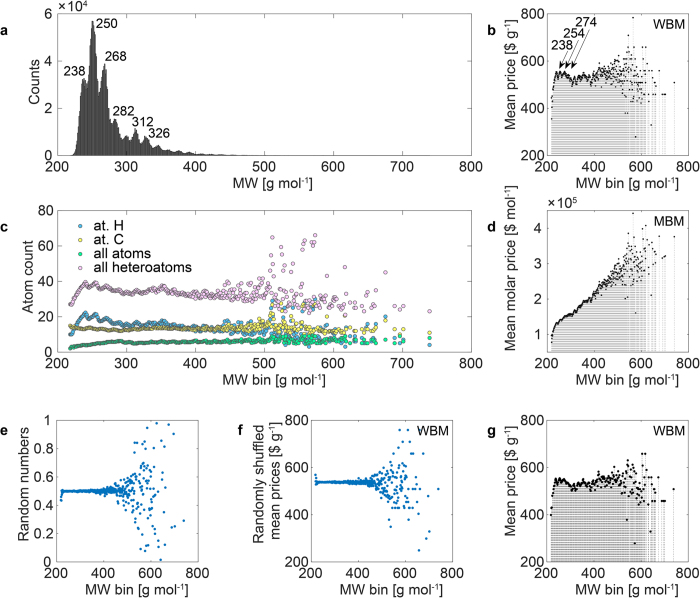
Binned MW statistics of chemical and economical data of the commercial library of ca. 2.2 mln compounds. (**a**) Frequency distribution of MWs of the MW between 218.26 to 738.74 Da. The majority of the compounds are below a MW of 350 (96%) or 400 Da (99%). (**b**) Mean price (WBM) vs. MW bins. (**c**) Average atom counts vs. MW bins. (**d**) Mean price (MBM) vs. MW bins. (**e**) Random numbers (0–1) and (**f**) randomly shuffled mean WBM prices; plotted vs. MW bins. In (**a–f**) data were rendered at a single Da MW unit. (**g**) Mean price (WBM) vs. MW bins with a rendering resolution of 2 Da units. The comparison of the binning mode in (**g vs. b**) does not reveal any substantial changes in the range below ca. 400 Da.

The MW of the molecules within the analyzed library ranges from 218 to 739 Da. Interestingly, within the MW frequency distribution (Fig. 2a) we observe a series of sharp maxima with the global one at ca. 250 Da which corresponds well to the molecular population of the Beilstein database reaction products^1^.

In Table 1 we analyzed the variability of the investigated building block library by the correlation matrix between the WBM and MBM prices and molecular descriptors, such as MW, atom counts, which can easily be computed from the molecular structures, and more refined values such as synthetic accessibility scores by the SYLVIA program^3^,^4^. The correlation between economic data and the individual molecular descriptors is rather poor. The maximum R coefficient is 0.47 and refers to WBM prices with MW.

Data binning or bucketing is a method in which the *original* [continuous] *data values which fall into a given small interval, a bin,* is *replaced by a value representative of that interval*. In Fig. 2b,d,g we plotted the WBM and MBM prices against the MW bins. This provided a surprisingly informative statistics up to ca. 400 Da. If we would like to answer the question of whether we are paying for the number of molecules or the size of the molecules in the WBM, then we can conclude that only within the lower MWs of up to about 250 Da the answer can be clear: the mean price increases with an increase in MW.

In Fig. 2d we plotted the mean molar prices against MW bins. The correlation coefficient for the relationship of mean molar price against MW bins amounts to R_bin_ = 0.93. In the context of economy, the relative high correlation between the price and MW needs a special explanation. In fact, with an increase of MW we need to buy a much larger amount of the substance to obtain the same number of molecules. For example, to buy a mole of benzene we need 78 g while for a mole of [18]annulene we need three times as much, 3^*^78 g. Thus, the correlation observed indicates on average it is the quantity of matter what we are paying for. Next, what we observe is the coincidence of the maxima within the frequency histograms (Fig. 2a) and mean price plots (Fig. 2b,d,g). In Fig. 2c we plotted the average atom counts against MW bins of the library. We can see that, as expected, putting together mean H, C and heteroatom counts results in the mean total atom count. Interestingly, we now also observe a plot that is evidently related to the frequency histogram of Fig. 2a with a similar structure of the maxima. Moreover, the total atom counts correlate well with the hydrogen counts which is illustrated by the similar shape of the corresponding plots. The correlation between non-binned data amounts to R = 0.983 (H vs. all) and 0.598 (C vs. all). We tested the statistical credibility of binning by a series of additional simulations. Thus, in Fig. 2e,f we present the relationship between the MW bins and randomly generated numbers or the randomly shuffled WBM prices, while in Fig. 2g the influence of the binning resolution is tested (compare section Methods for details).

We carefully investigated the influence of the C, H and heteroatom counts on mean prices (compare supplementary materials). An interesting observation is that there are two types of heteroatoms here. On average, N, O, S and F atoms, contribute in similar manner to the price as the C content, but are more expensive than Cl, Br, I or Se atoms (WBM). Moreover, quite unexpectedly, the mean prices generally drop with an increase in the number of heteroatoms especially for the second group of atoms (Cl, Br, I or Se). Nitrogen is the only exception here as an increase in the number of N-atoms results in a higher price. We can offer two explanations for this effect. First unlike N, O, S atoms, which most often are contained in the synthesized scaffolds, the Cl, Br or I atoms are generally used to bring reactive functionality into the synthons. Thus, although their presence is absolutely necessary, they are removed in reactions, therefore, producing the lower MW targets. Thus, the use of such functions is highly uneconomic. Although we are well aware of these losses in chemistry, the term of *atom economy* has appeared in chemistry relatively recently, and has inspired searches for novel chemistries capable of providing better atom economy efficiency^5^. Even if such a scenario seems attractive, the explanation may be much simpler. Thus, heteroatoms are heavier than carbon atoms. This means that if we include them in the products we are offering on average within the same weight unit a lower number of (carbon) atoms for the construction of the chemical skeleton. This also means that the targets with heteroatoms should have a lower total number of atoms in comparison with the molecules having approximately the same MW values or more precisely, located in the same MW bins (iso-MW bins) while generated exclusively from carbon. Can we observe this effect more clearly and does it really matter in substance pricing?

**Figure 3:**
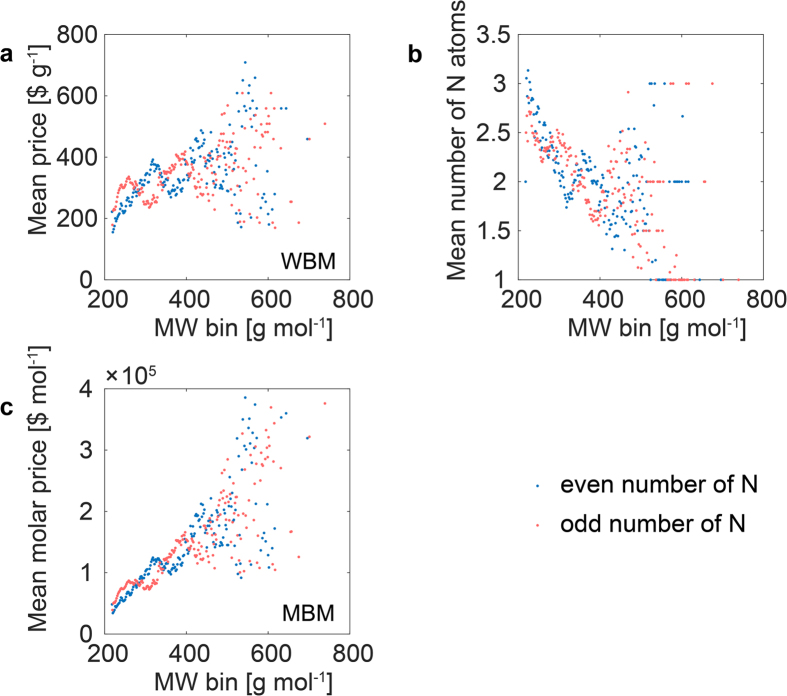
Binned MW statistics for the nitrogen sub-library. (**a**) Mean price (WBM) vs. MW bins; (**b**) mean number of nitrogen atoms in a molecule plotted against MW bins and (**c**) mean price (MBM) plotted against MW bins.

To answer this question it will be interesting to test the heteroatom sub-libraries. In Fig. 3 we show the WBM mean price statistics for the nitrogen atom sub-library. We should remember that we are now comparing the mean price for compounds within iso-MW bins. In the case of N we are observing an especially interesting effect. The so-called nitrogen rule decides that for compounds consisting of only C, H, O and N atoms an odd or an even number of N atoms in a molecule having an odd or even number of MW is generated, respectively. We can see in Fig. 3a,b that, quite unexpectedly, these groups are priced differently. Moreover, there is no regularity which group is cheaper and which one is more expensive. The higher pricing changes with the MW range. However, when we compare Fig. 3a,b to Fig. 3c where we plotted the mean number of N atoms vs. MW bins we can see that the larger mean number of N always results in a lower average price of the products. This proves that the number of atoms (atom count) influences the price of iso-MWs. To hypothesize a possible explanation of this effect we should follow chemistry. The larger atomic weight of N vs. C (14 Da vs. 12 Da) decides that if we include several N atoms within the structure we are decreasing the total number of atoms (atom counts per molecule) in comparison with their C iso-MW bins. As a consequence, lower total atom counts per MBM are offered. The difference in the C and N valence further decides that the C and N iso-MWs differ in the number of atom counts. Can this decide the price?

## Discussion

Probing large molecular populations differs from a classical QSAR approach investigating much smaller data sets in that observables describing individual compounds are often replaced by mean, maximum or minimum values describing larger compound series or classes. For example, in the Lipinski rule of 5, probably the best known approach of this type, the maximum values of molecular weights or logPs were used to identify a class of drugs. In other words, what we are doing here is probing all real drugs on the market and then using the typical values of several molecular descriptors for this class to guide the search for new drug candidates. Data binning is an option that can replace the mapping of median or mean values, while histograms or counts are typical visualization for such a data type and examples can be found in reference^6^. We can call all the above mentioned approaches *molecular statistics,* which can be defined as a massive probing of the formal structure of chemical space, by sampling chemical compounds to measure the statistics of molecular descriptors and/or properties. Obviously, molecular statistics is a type of SAR, but the difference is that we focus here more on the compound classes that are more fuzzy than on individual drugs or chemotypes, e.g., all drugs, non-drugs, FDA approvals, etc. On the other hand, we are analyzing here much larger molecular populations in the hope of identifying the domains of the chemical space specific to the new classes and the rules that control these domains. Other examples of molecular statistics can be found in references^7^,^8^,^9^,^10^. Here we are reporting a molecular statistics in which, in particular, the compound classes are binned MWs (iso-MWs), which can formally be formed of very different structures.

**Figure 4:**
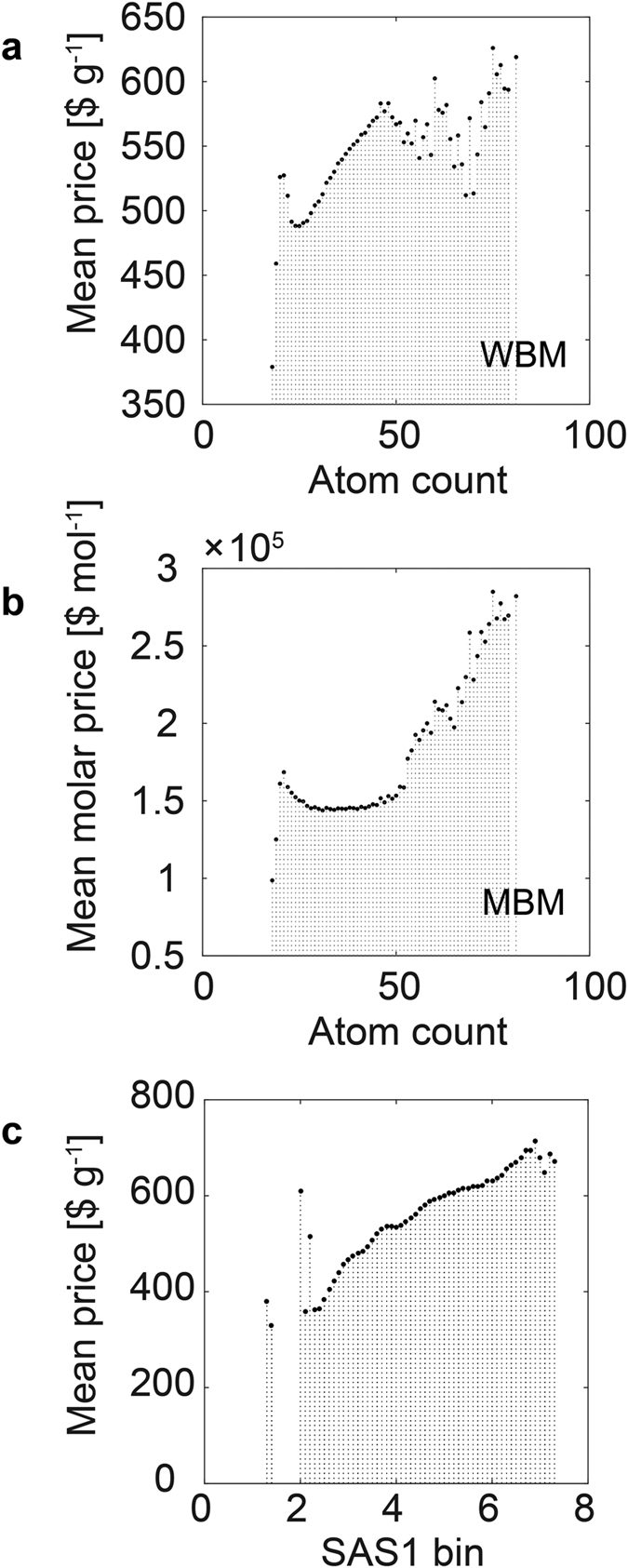
Mean prices vs. mean atom counts. (**a**) WBM; (**b**) MBM and (**c**) bin SAS1 statistics.

For large data it is hard to expect that we can observe a precise quantitative relationships as expected for QSAR models for small datasets. In fact, the correlations between economic data and different molecular descriptors are relatively low (Table 1). Data binning allowed us to observe clear trends. If we compare the atom counts plots with the price plots we can see the outstanding resemblance between the prices (Fig. 2b) and the total atom counts but not carbon counts (Fig. 2c). Because molecular graphs are often only represented by the carbon/heteroatom structures we should conclude that a simple molecular formula (MF) is more important for pricing than the full molecular graph. The molecular market is an expert bazaar. Then one of the simplest measure of molecular complexity available on a low-level expertise is the atom count, which shapes the size and visual representation of the molecule. Without deeper analyses, the molecules with the lower total atom count looks to the chemists less complex than those with a higher atom count. In the language of economy they are less attractive than higher atom count MWs. This answers the question how the atom count can influence a price. Interestingly, if we would like to transform this information fully properly, i.e., preserving its chemical meaning, into the pricing we should have done this in the MBM. Apparently, however, prices are given in WBM and this effect imprints on the WBM, tracing also the fact that pricing has been truly performed in WBM and not MBM (Fig. 4a vs 4b). Finally, we can see in Fig. 4c that chemistry plays a role in pricing and the mean WBM price depends on the binned synthetic accessibility score SAS1^3^,^4^ of the library (R_bin_ = 0.925).

The reported statistics relates the prices of the chemicals to molecular descriptors, therefore, focusing on chemical indicators. However, as pointed out by a reviewer, also the use of a chemical has a strong influence on the price. Accordingly, further studies are needed to include this effect into a fully predictive QSER model.

## Methods

The catalogue data of a large library of 2,248,243 chemicals offered on the market were downloaded from the internet site (http://www.abamachem.net/). The data were delivered in the SDF format. Next, the records were carefully inspected, cured^11^ and prepared for further processing, e.g., duplicated notations were removed. Instant JChem version 14.7.28.0 released in 2014^12^ and additional self-programmed scripts were used for structure database management. Calculations were performed using MATLAB version R2015b on an Intel Core 2 Duo CPU 2.20 GHz computer system with 8.00 GB RAM and a 64-bit Windows operating system. The synthetic accessibility scores (Table 1, SAS) were calculated using SYLVIA version 1.4 (Molecular Networks GmbH)^3^,^4^. All but 180 records were successfully processed to their respective SYLVIA scores.

The statistical credibility of the MW binning method was tested in a series of additional simulations to avoid chance effects of binning^13^. Thus, in Fig. 2e,f we presented the relationship between the MW bins and randomly generated numbers or the randomly shuffled prices (WBM). In Fig. 2e,f we see that within these MW bins that are filled with the large number of molecules (below ca. 400 Da) the calculated mean outputs of the bins are very stable taking a mean value of the original variable (random numbers between 0 and 1 give a mean of 0.5 while the randomly shuffled WBM prices take approximately a mean value of the prices which is 537.83 $). However, if we go to the bins occupied by a smaller number of molecules (MW over 400 Da) we observe large fluctuations in both plots (Fig. 2e,f). Moreover, changing the binning resolution (the number of MW bins) does not significantly change the plot as can be compared in Fig. 2b vs. 2 g.

Correlation coefficient evaluated for 10000 random runs indicated for the original non binned data a mean absolute value of R^random^ = 0.000526 (random numbers) or R^shuffled^ = 0.000530 (randomly shuffled prices) which are at least three orders lower than the R data given in Table 1. For the binning experiment we repeated similar simulations 2000 times and averaged the absolute R values to obtain the mean correlation value of R_bin_^shuffled^ amounting to 0.06168 (mean MBM prices vs binned MW) or 0.05748 (mean WBM prices vs binned MW).

## Additional Information

**How to cite this article**: Polanski, J. *et al*. Molecular descriptor data explain market prices of a large commercial chemical compound library. *Sci. Rep.* **6**, 28521; doi: 10.1038/srep28521 (2016).

## Supplementary Material

Supplementary Information

## Figures and Tables

**Table 1:** Correlation coefficients between molecular descriptors, WBM and MBM prices^a^.

	P1^b^	P2^c^	MW^d^	AC^e^	SAS1^f^
P1	1	0.857	−0.033	0.171	0.341
P2	0.857	1	0.474	0.045	0.239
MW	−0.033	0.474	1	−0.213	−0.116
AC	0.171	0.045	−0.213	1	0.380
SAS1	0.341	0.239	−0.116	0.380	1

^a^Statistical credibility tests indicate much lower correlation values (R = 0.000526) for the shuffled price values, compare Methods an supplementary materials for additional data.

^b^P1 - Price (WBM).

^c^P2 - Molar price (MBM).

^d^MW - molecular weight.

^e^AC - Atom count.

^f^SAS1 - synthetic accessibility score.
