# Supplementary material for: Molecular descriptor data explain market prices of a large commercial chemical compound library
Source: Sci Rep. 2016 Jun 23;6:28521. doi: 10.1038/srep28521 (PMC4917835; doi:10.1038/srep28521)
Supplement: Supplementary Information [file srep28521-s1.pdf]

Molecular descriptor data explain market prices of a large commercial chemical compound library

Jaroslaw Polanski,*[a] Urszula Kucia,[a] Roksana Duszkiewicz,[a] Agata Kurczyk,[b] Tomasz Magdziarz,[b] Johann Gasteiger[c]

1. *Institute of Chemistry, University of Silesia, 9 Szkolna Street, 40-006 Katowice, Poland*

2. *Institute of Automatic Control, Silesian University of Technology, 16 Akademicka Street, 44-100 Gliwice, Poland*

3. *Silesian University of Technology, 16 Akademicka Street, 44-100 Gliwice, Poland*

4. *Computer-Chemie-Centrum, University of Erlangen-Nuernberg, Naegelsbachstrasse 25, 91052 Erlangen, Germany.*

Table of Contents

Data 3
Variability of the investigated building block library by the correlation matrix 4
Statistical analyses 5
Molecular descriptors and data preprocesing...5
Principal component analysis (PCA) 6
Additional figures..8
....

Data

Since data exceed the allowed upload volume the excel files (ca. 50 MB) with all data and calculated SYLVIA scores together with the scripts are available on request from authors.

Variability of the investigated building block library by the correlation matrix

Full version of Table 1.

Table S1 Correlation coefficients for simple molecular descriptors, MW, atom counts, and calculated synthetic availability indicators, in assessing WBM and MBM prices of the building block library of 2,248,243 compounds

	P1	P2	MW	AC	C	H	N	SAS1	SAS2	SAS3	SAS4	SAS5
P1	1	.857	-.033	.171	-.105	.177	.216	.341	.006	-.001	.200	.157
P2	.857	1	.474	.045	-.108	.005	.096	.239	.168	-.004	.118	.113
MW	-.033	.474	1	-.213	-.034	-.302	-.187	-.116	.323	-.007	-.113	-.053
AC	.171	.045	-.213	1	.598	.983	-.102	.380	-.245	-.160	.403	-.103
C	-.105	-.108	-.034	.598	1	.542	-.446	.081	-.212	.115	.215	-.117
H	.177	.005	-.302	.983	.542	1	-.093	.378	-.349	-.169	.421	-.100
N	.216	.096	-.187	-.102	-.446	-.098	1	.031	.049	.019	-.175	.345
SAS1	.341	.239	-.116	.380	.081	.378	.031	1	.173	.182	.831	.206
SAS2	.006	.168	.323	-.245	-.212	-.349	.049	.173	1	.097	-.079	.114
SAS3	-.001	-.004	-.007	-.160	.115	-.169	.019	.182	.097	1	.015	.199
SAS4	.200	.118	-.123	.403	.215	.421	-.175	.831	-.079	.015	1	-.026
SAS5	.157	.113	-.053	-.103	-.117	-.100	.345	.206	.114	.199	-.026	1

The synthetic accessibility scores (SAS1-SAS5) were calculated using SYLVIA software; all correlation coefficients were calculated by MATLAB *corrcoef* function including formally discrete atom counts variables.

P1 - Price (WBM);
P2 - Molar price (MBM);
MW -molecular weight;
AC - Atom count;
C - C atom count;
H - H atom count;
N - N atom count;
SAS1 - synthetic accessibility score (M_SYN_ACCESSIBILITY);
SAS2 - molecular graph complexity score (M_GRAPH_SCORE);
SAS3 - ring complexity score (M_RING_SCORE);
SAS4 - stereochemical complexity score (M_STEREO_SCORE);
SAS5 - reaction center substructure score (M_REACTION_CENTER_SCORE).

Statistical analyses

A principal component analysis (PCA) if used to analyze the data variability indicates that the first two components explain only 30% of variance.

Molecular descriptors and data preprocesing

Following is a list of molecular descriptors used in PCA analysis:

- Mol_weight
- Price_1g
- LogP
- Atom_count
- Bond_count
- Chiral_atom
- H_bond_acteptors
- H_bond_donors
- Ring_count
- Rotable_bonds
- C
- H
- N
- O
- S
- Br
- F
- I
- Se
- Na
- Sn
- P
- Si
- B
- K
- Cl

Additionally following descriptors calculated by RDKit 201403 were used:

- BalabanJ
- BertzCT
- FractionCSP3
- HallKierAlpha
- HeavyAtomCount
- HeavyAtomMolWt
- Ipc
- Kappa1
- Kappa2
- Kappa3
- LabuteASA
- MaxAbsEStateIndex
- MaxEStateIndex
- MinAbsEStateIndex
- MinEStateIndex
- MolMR
- NHOHCount

- NOCount
- NumAliphaticCarbocycles
- NumAliphaticHeterocycles
- NumAliphaticRings
- NumAromaticCarbocycles
- NumAromaticHeterocycles
- NumAromaticRings
- NumHeteroatoms
- NumRotatableBonds
- NumSaturatedCarbocycles
- NumSaturatedHeterocycles
- NumSaturatedRings
- NumValenceElectrons
- TPSA

Additionally 5 descriptors calculated by SYLVIA were used

- M_SYN_ACCESSIBILITY
- M_GRAPH_SCORE
- M_RING_SCORE
- M_STEREO_SCORE
- M_REACTION_CENTER_SCORE

In total 62 molecular descriptors were used. Molecular descriptors calculations failed for 182 compounds (RDKit failed for 2, SYLVIA failed for 180) and these compounds were removed form the set. Resulting matrix was of shape 2248061 x 62.
Prior to PCA data was submitted to so called Standardization preprocesing, ie columns were centralized (mean values equal to 0) and divided by standard deviation values (variance set to 1 for all columns).

Principal component analysis (PCA)

A principal component analysis (PCA) if used to analyze the data variability indicates that the first two components explain only 30% of variance and 53 components explain 100% (see also Figure S2). Figure S1 shows score plot of PC1 and PC2. Top ten contributing variables for first two components is following:

- TPSA
- H
- Atom_count
- NumHeteroatoms
- FractionCSP3
- Bond_count
- NOCount
- NumAromaticRings
- BertzCT
- C

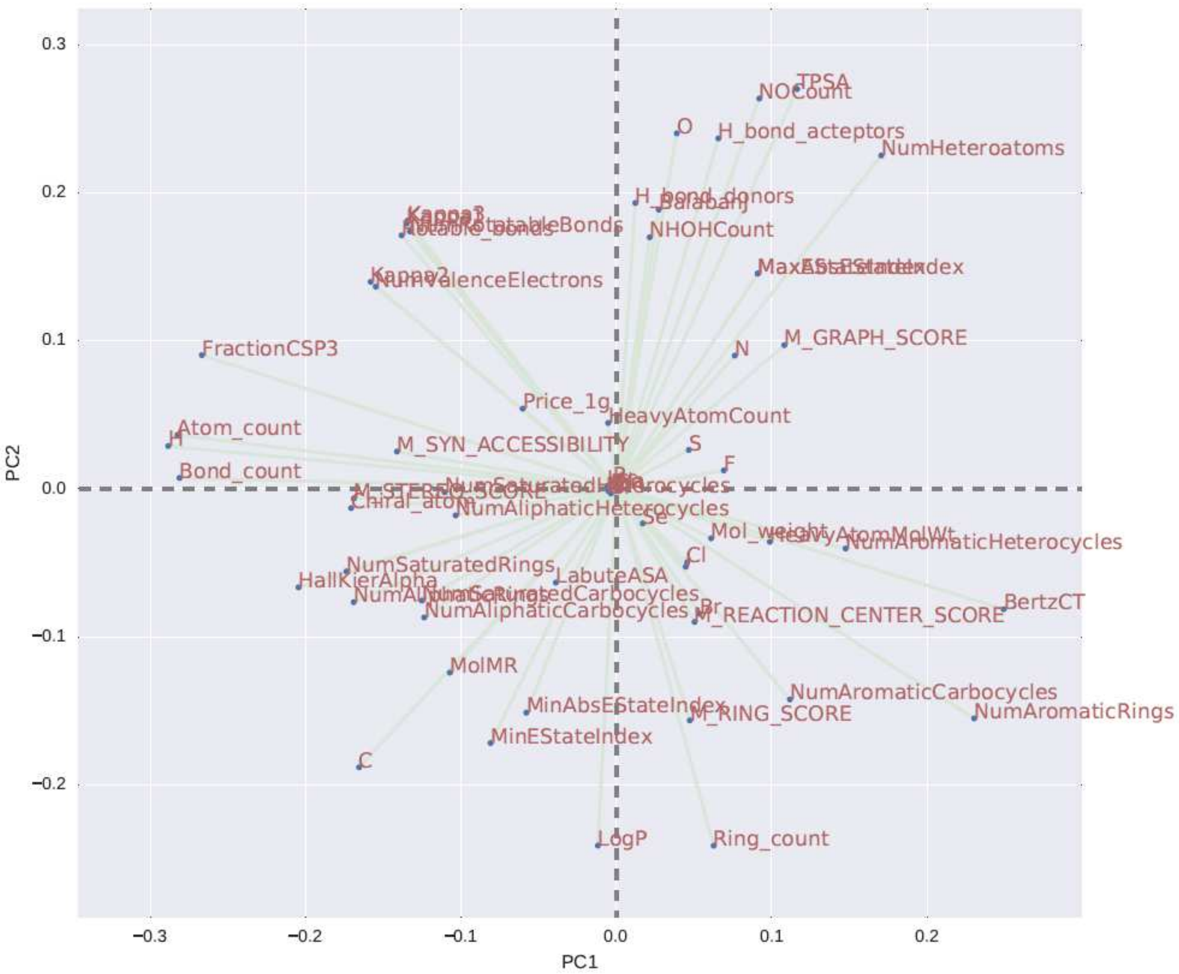

Figure S1. PCA score plot, projection on PC1 and PC2.

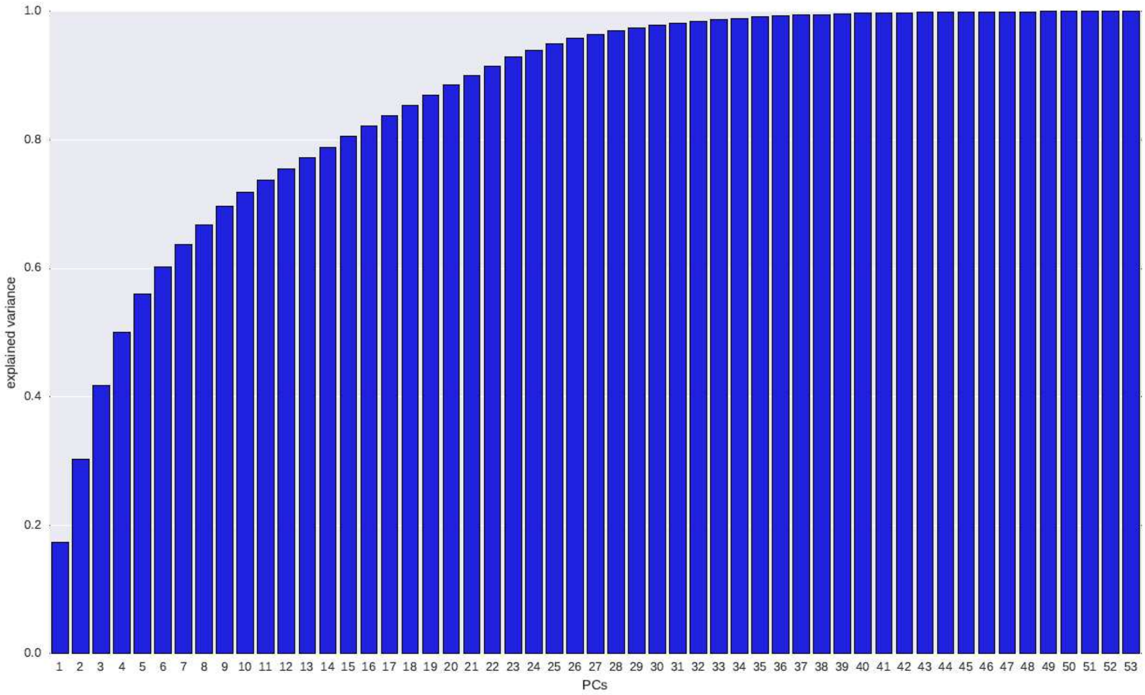

Figure S2. Cumulative variance up to first 53 principal components.

Additional figures

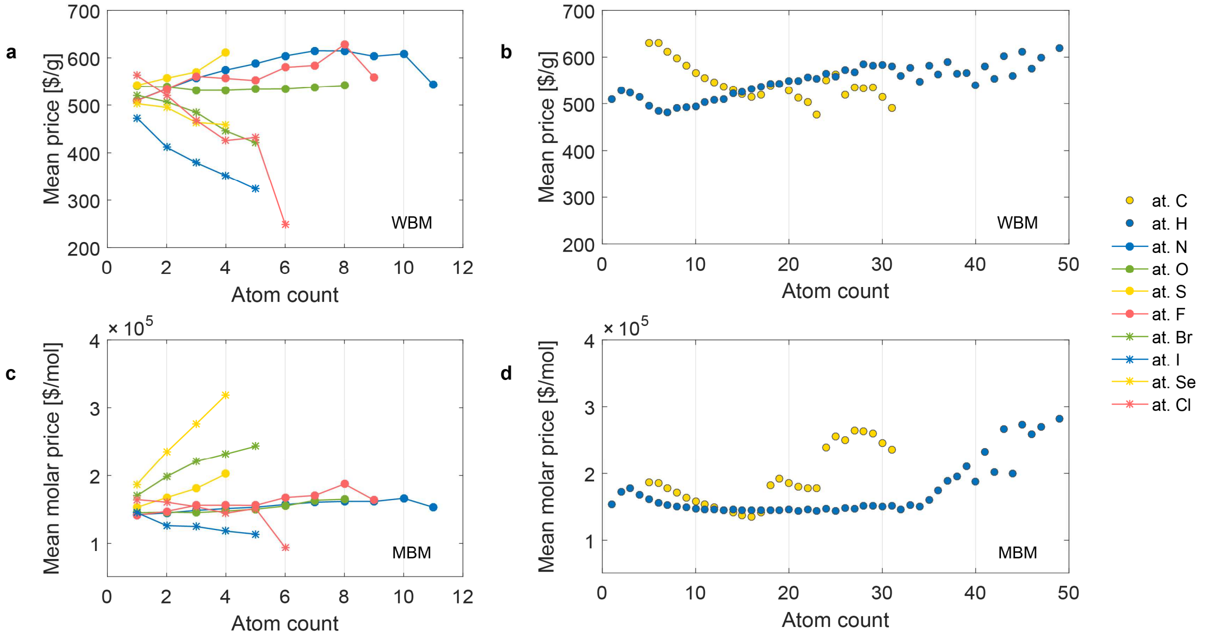

Figure S3. Atom count statistics of economic data. a-b, Mean prices (WBM) and **c-d,** Mean molar prices (MBM) plotted vs. (hetero)atom count.

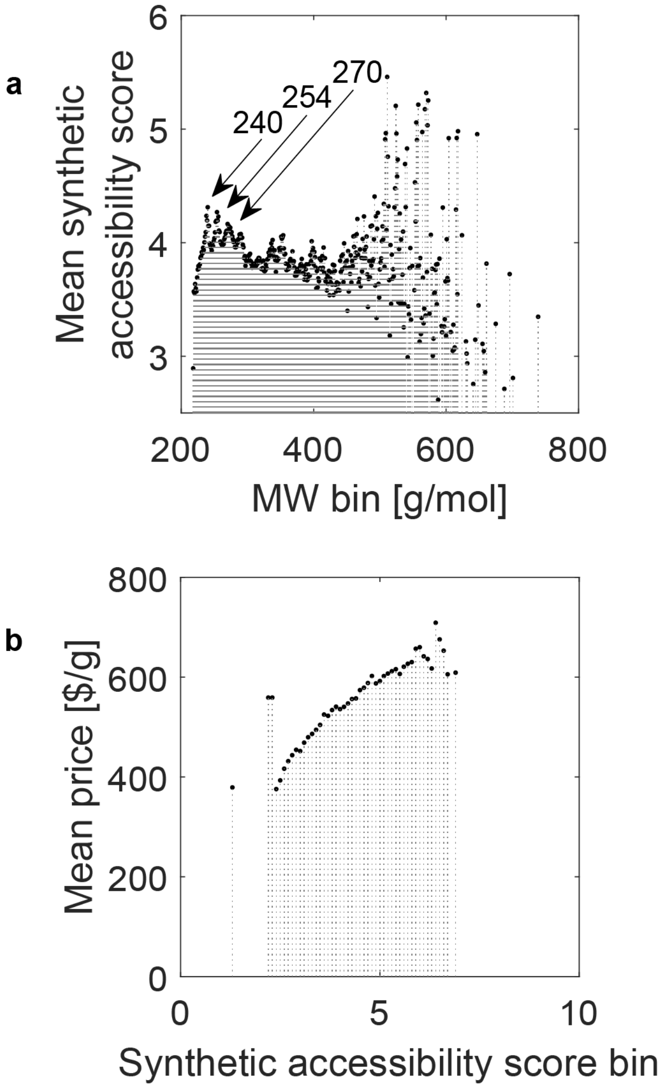

Figure S4. a, Binned MW statistics of mean synthetic accessibility (SYLVIA) compared with **b,** Mean prices (WBM) plotted as a function of synthetic accessibility bins.

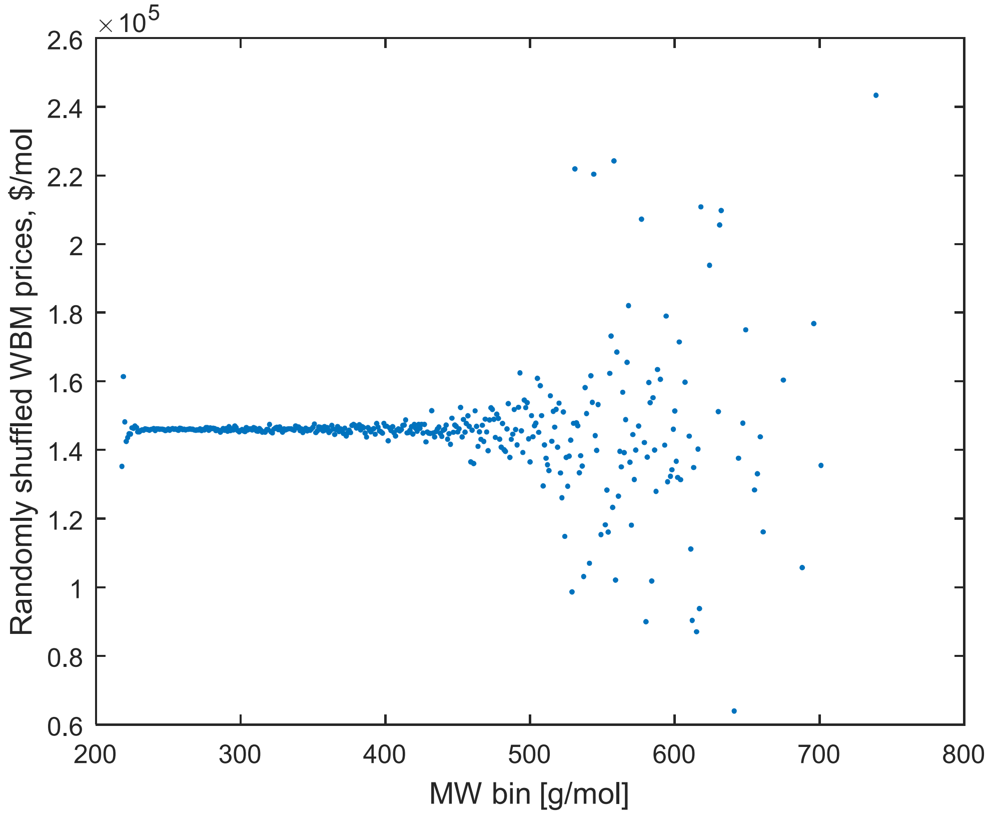

a

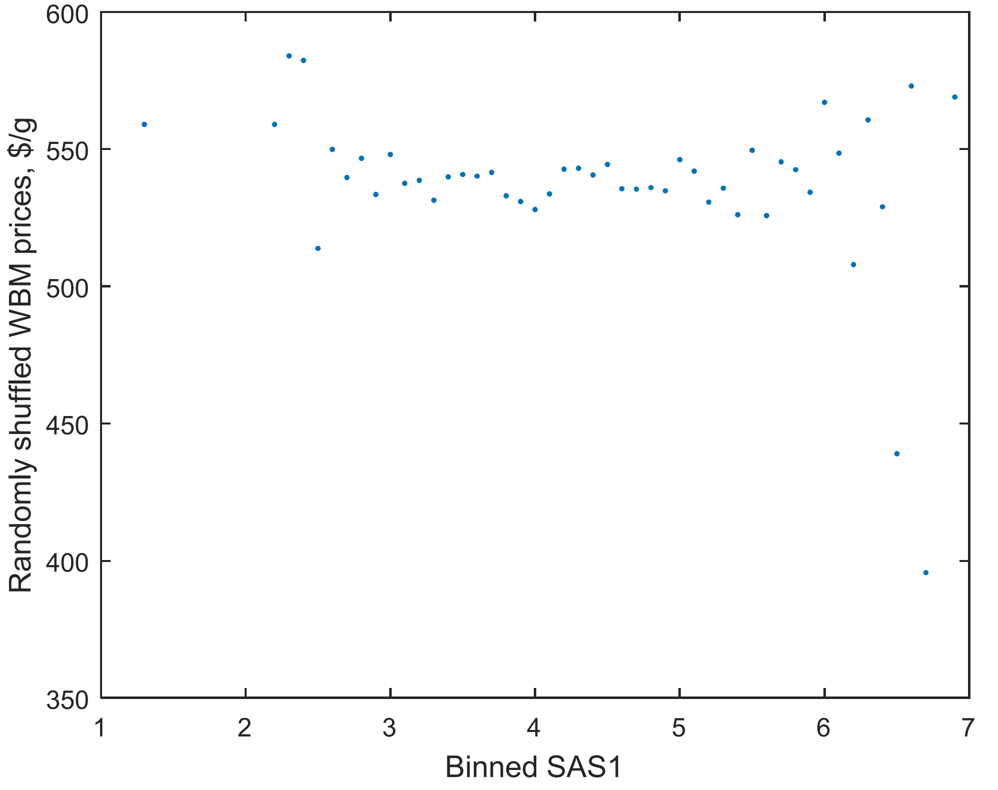

b

Figure S5. Statistical credibility tests. a, randomly shuffled mean WBM prices plotted vs. MW bins and random numbers (0-1); **b.** randomly shuffled mean WBM prices plotted vs. binned SAS1; mean value of MBM price amounts to 1.45 * 10^5 $/mol.
